# Occult Malignancy Presenting as Pericardial Effusion: The Diagnostic Contribution of Pericardial Fluid Cytology

**DOI:** 10.3390/cancers18203368

**Published:** 2026-10-18

**Authors:** Iulia Raluca Munteanu, Ramona Cristina Novaconi, Radu Ioan Lala, Caius Glad Streian, Ciprian Nicușor Dima, Adrian Petru Merce, Lucian Silviu Falniță, Marinela-Adela Scuturoiu, Adrian-Grigore Merce, Alina Gabriela Negru, Horea Bogdan Feier

**Affiliations:** 1 Doctoral School Medicine-Pharmacy, “Victor Babes” University of Medicine and Pharmacy Timisoara, E. Murgu Sq. No. 2, 300041 Timisoara, Romania; iulia.munteanu@umft.ro (I.R.M.); adrian-grigore.merce@umft.ro (A.-G.M.); 2 Clinic of Cardiovascular Surgery, Institute for Cardiovascular Diseases of Timisoara, Gheorghe Adam Street, No. 13A, 300310 Timisoara, Romania; streian.caius@umft.ro (C.G.S.); dima.ciprian@umft.ro (C.N.D.); adimerce@gmail.com (A.P.M.); lfalnita@gmail.com (L.S.F.); horea.feier@umft.ro (H.B.F.); 3 Advanced Research Center, Institute for Cardiovascular Diseases, 300310 Timisoara, Romania; adela.scuturoiu@umft.ro; 4 Multidisciplinary Doctoral School, Vasile Goldis Western University of Arad, 310025 Arad, Romania; 5 Victor Babes Clinical Hospital for Infectious Diseases and Pneumology of Timisoara, 300041 Timisoara, Romania; 6 Department VI Cardiology, Cardiovascular Surgery Clinic, “Victor Babes” University of Medicine and Pharmacy Timisoara, E. Murgu Sq. No. 2, 300041 Timisoara, Romania; 7 NEUROPSY-COG Center for Cognitive Research in Neuropsychiatric Pathology, Department of Neuroscience, “Victor Babes” University of Medicine and Pharmacy, 300041 Timisoara, Romania; 8 Department of Cardiology, “Victor Babes” University of Medicine and Pharmacy, E. Murgu Sq. No. 2, 300041 Timisoara, Romania; alinanegru@umft.ro

**Keywords:** pericardial effusion, pericardial fluid cytology, malignant pericardial effusion, occult malignancy, cardiac tamponade, cardio-oncology

## Abstract

**Background/Objectives:** Pericardial effusion may occur in patients with established cancer but may also represent the first manifestation of an occult malignancy. This study evaluated the diagnostic contribution of pericardial fluid cytology and pathological assessment according to previous oncological status, with particular emphasis on previously unrecognized malignancy. **Methods:** We conducted a retrospective observational study of patients hospitalized with pericardial disease between January 2021 and December 2025 at a tertiary cardiovascular center. The final cohort comprised 234 clinical episodes in 209 patients. Clinical, laboratory, imaging, procedural, cytological, and histopathological data were analyzed in relation to previous oncological status and malignant pericardial involvement. **Results:** Pathological assessment was available in 95/234 episodes (40.6%), with neoplastic involvement identified in 21/95 (22.1%). Neoplastic findings occurred in 13/33 (39.4%) evaluable episodes with known malignancy and 8/62 (12.9%) without a previous cancer diagnosis (odds ratio, 4.39; 95% confidence interval, 1.59–12.11; *p* = 0.004). Conversely, 20/33 (60.6%) evaluable episodes in patients with known cancer showed no neoplastic involvement. In all eight patients without previously known malignancy, pericardial fluid cytology provided the first pathological evidence of cancer; six (75%) had findings suggestive of a pulmonary origin. All presented with large effusions and hemodynamic compromise. **Conclusions:** Pericardial pathological assessment provides diagnostic information beyond previous oncological status. It can distinguish malignant from non-neoplastic pericardial involvement in patients with established cancer while also revealing occult malignancy when pericardial disease is its first presentation.

## Simple Summary

Pericardial effusion may occur in patients with cancer but does not necessarily indicate malignant involvement. It may also be the first manifestation of an otherwise unknown malignancy. This study investigated whether examination of pericardial fluid can help distinguish these situations. Many patients with known cancer had no malignant involvement, whereas in eight patients without a previous cancer diagnosis, pericardial fluid cytology provided the first pathological evidence of malignancy, most often of pulmonary origin. These findings highlight the diagnostic value of pericardial fluid cytology beyond previous oncological history and its potential role in revealing occult malignancy.

## 1. Introduction

Pericardial effusion remains a challenging clinical entity, not because of the technical difficulties associated with its detection, but because of the remarkable diversity of diseases capable of producing an apparently similar presentation. While contemporary imaging techniques have substantially improved the recognition and anatomical characterization of pericardial fluid collections, establishing the underlying etiology continues to represent the cornerstone of patient evaluation [1]. Indeed, the presence of pericardial fluid should be interpreted as the consequence of an ongoing pathological process rather than as a diagnosis itself, requiring careful integration of clinical, laboratory, imaging, and pathological findings to guide subsequent management [2].

Among the broad spectrum of etiologies, malignant disease occupies a unique position because of its diagnostic complexity, prognostic implications, and therapeutic consequences [3]. Pericardial involvement may occur at any stage of the oncological disease course, occasionally constituting its first clinical manifestation and posing significant diagnostic challenges even in the era of advanced multimodality imaging [4]. Consequently, the identification of the underlying cause of pericardial effusion extends beyond determining its origin, directly influencing treatment strategies, oncological evaluation, and patient outcomes [5].

### 1.1. Pericardial Effusion: Etiological Complexity and Clinical Significance

Pericardial effusion should not be regarded as a distinct disease entity but rather as a common clinical manifestation shared by a broad spectrum of cardiovascular and systemic disorders. Although patients frequently present with similar clinical and echocardiographic findings, the underlying pathological processes responsible for pericardial fluid accumulation differ substantially in their pathophysiology, biological behavior, natural history, and therapeutic implications [6,7]. Pericardial fluid accumulates whenever the physiological equilibrium between its production and resorption is disrupted, a process that may result from diverse mechanisms including inflammation, infection, autoimmune disease, metabolic disorders, trauma, medical interventions, or neoplastic involvement [8]. Consequently, pericardial effusion represents the final common pathway of numerous diseases rather than a diagnosis in itself.

This etiological heterogeneity is mirrored by an equally diverse clinical spectrum. The hemodynamic impact of a pericardial effusion depends not only on the absolute volume of accumulated fluid, but also on the rate of its accumulation, the mechanical properties of the pericardium, and the patient’s cardiovascular reserve [9]. As a result, comparable effusion volumes may be associated with markedly different clinical scenarios, ranging from incidental asymptomatic findings to rapidly progressive cardiac tamponade with severe circulatory compromise [10]. Likewise, similar clinical presentations may conceal fundamentally different underlying diseases, each requiring a distinct diagnostic approach and disease-specific management.

### 1.2. Pericardial Involvement in Malignant Disease

Among the diverse etiologies of pericardial effusion, malignant disease represents a distinct clinical entity because of its unique biological behavior, profound therapeutic implications, and consistently unfavorable prognosis [11]. Although primary tumors of the pericardium are exceptionally uncommon, secondary involvement resulting from metastatic dissemination accounts for the vast majority of neoplastic pericardial disease. Pericardial invasion may occur through direct extension from adjacent thoracic malignancies, lymphatic spread, hematogenous dissemination, or, less frequently, by transvenous extension [3]. These mechanisms reflect the complex interactions between tumor biology and the pericardial microenvironment, ultimately leading to inflammation, impaired lymphatic drainage, increased vascular permeability, or direct neoplastic infiltration, all of which may contribute to pericardial fluid accumulation [4].

The clinical spectrum of malignant pericardial involvement is remarkably heterogeneous. Patients may remain asymptomatic despite extensive pericardial infiltration, whereas others present with rapidly progressive pericardial effusion, cardiac tamponade, or recurrent fluid accumulation requiring repeated intervention [12]. Importantly, pericardial involvement does not necessarily occur exclusively in advanced stages of cancer. In a subset of patients, pericardial effusion represents the earliest clinically evident manifestation of an otherwise occult malignancy, preceding the diagnosis of the primary tumor and initiating the subsequent oncological work-up [13,14]. Such presentations pose a particular diagnostic challenge, as neither clinical manifestations nor conventional imaging findings are sufficiently specific to reliably distinguish malignant from non-malignant etiologies [3].

Beyond its immediate hemodynamic consequences, malignant pericardial involvement has important implications for disease staging, therapeutic planning, and overall prognosis [5]. Consequently, establishing the underlying cause of pericardial effusion is of particular importance whenever malignancy is suspected, as timely recognition of neoplastic disease may substantially influence subsequent clinical management.

### 1.3. Contemporary Diagnostic Evaluation of Pericardial Effusion

The diagnostic evaluation of pericardial effusion requires a comprehensive and sequential approach aimed not only at confirming the presence of pericardial fluid, but also at identifying the pathological process responsible for its development. Initial assessment relies on clinical history, physical examination, laboratory investigations, and multimodality imaging, each contributing complementary information to the overall diagnostic framework [2]. However, because pericardial effusion represents a common manifestation of multiple diseases rather than a specific pathological entity, no single diagnostic modality is capable of establishing its etiology in every clinical scenario.

Transthoracic echocardiography remains the first-line imaging modality owing to its widespread availability, real-time assessment of effusion size, distribution, and hemodynamic significance, as well as its ability to identify features suggestive of cardiac tamponade [15]. Cross-sectional imaging with computed tomography and cardiac magnetic resonance further expands diagnostic capabilities by providing superior anatomical definition of the pericardium and adjacent thoracic structures, facilitating the identification of pericardial thickening, nodularity, extracardiac malignancy, mediastinal disease, or associated intrathoracic pathology. In selected patients, positron emission tomography may provide additional functional information by identifying metabolically active lesions suggestive of neoplastic or inflammatory disease [1,7].

Despite these advances, imaging techniques primarily characterize the anatomical and functional consequences of pericardial disease rather than definitively establish its underlying etiology. Clinical presentation and imaging findings frequently overlap among inflammatory, infectious, autoimmune, and neoplastic disorders, limiting their diagnostic specificity [16]. Consequently, etiological diagnosis often requires integration of imaging findings with laboratory investigations and direct analysis of pericardial fluid or tissue whenever clinically indicated [2].

Within this multidisciplinary diagnostic framework, pericardial fluid analysis remains particularly important when the underlying etiology is uncertain or malignancy is suspected [3].

### 1.4. Pericardial Fluid Cytology: Current Evidence and Unresolved Challenges

Despite substantial advances in multimodality imaging, establishing the definitive etiology of pericardial effusion frequently requires pathological confirmation. Whenever pericardial fluid is obtained for therapeutic or diagnostic purposes, its laboratory evaluation represents a unique opportunity to investigate the underlying disease process. In routine clinical practice, biochemical, microbiological, and cytological analyses provide complementary information, while histopathological examination of pericardial tissue may further contribute when tissue samples are available [17]. Among these investigations, cytological examination occupies a particularly important position because it directly evaluates the cellular composition of the effusion and may provide evidence of malignant pericardial involvement [18].

The diagnostic performance of pericardial fluid cytology has been extensively investigated; however, reported results remain heterogeneous [17]. Variability in patient selection, underlying disease, sampling techniques, specimen processing, and cytopathological interpretation has contributed to considerable differences in the reported diagnostic yield across published studies [19]. Although previous studies have examined the diagnostic performance of pericardial fluid cytology, its contribution within the clinical pathway deserves separate consideration, particularly when malignant pericardial involvement represents the first manifestation of an otherwise unrecognized cancer [20].

From a clinical perspective, the value of pericardial cytology extends beyond confirming malignant involvement in patients with an already established oncological diagnosis. In selected cases, malignant cells identified within the pericardial fluid may constitute the earliest objective evidence of an otherwise occult neoplasm, fundamentally altering subsequent diagnostic investigations, disease staging, and therapeutic planning [13]. Conversely, the diagnostic yield of cytological examination is unlikely to be uniform across different malignancies, reflecting the biological diversity of tumor dissemination and pericardial involvement [17,18,21]. Nevertheless, contemporary data addressing these clinically relevant aspects remain limited.

A better understanding of the diagnostic contribution of pericardial fluid cytology within the etiological assessment of pericardial effusion may therefore improve clinical decision-making and refine the diagnostic approach to patients in whom malignant disease is suspected.

## 2. Materials and Methods

A retrospective observational cohort study was conducted at the Institute of Cardiovascular Diseases Timișoara, Romania, that included all consecutive patients admitted to the Department of Cardiovascular Surgery between 1 January 2021 and 31 December 2025. The study was reported in accordance with the Strengthening the Reporting of Observational Studies in Epidemiology (STROBE) guidelines (Table S1) [22]. Eligible patients were identified through the institutional electronic medical record system by screening admissions to the Department of Cardiovascular Surgery with a primary or secondary discharge diagnosis coded as pericarditis. Cardiac tamponade was not identified through a separate diagnostic code but was identified during manual review of the diagnoses recorded in the discharge summaries. The initial search identified 332 hospitalizations, comprising 285 with a primary diagnosis and 47 with a secondary diagnosis of pericarditis.

To ensure accurate case identification and minimize potential selection bias, all medical records underwent independent manual review by two investigators. The screening process was repeated on three separate occasions, and any discrepancies regarding eligibility or etiological classification were resolved by consensus following comprehensive review of the complete clinical documentation. This verification process was implemented to ensure that only patients with true pericardial disease were included in the final study cohort.

Patients were eligible for inclusion if they were hospitalized during the predefined study period with a confirmed diagnosis of pericardial disease and had complete medical documentation available for review. Following manual review, 98 records were excluded. These comprised 11 cases of constrictive pericarditis, which were excluded because this entity represents a chronic fibro-inflammatory syndrome with distinct pathophysiological mechanisms, clinical evolution, and therapeutic management; 73 cases of cardiac rupture, acute postoperative cardiac tamponade, or post-pericardiotomy pericardial effusion occurring within the first two postoperative weeks, which were excluded because these conditions represent procedure-related complications rather than primary pericardial disease; and 14 duplicate records referring to the same hospitalization.

**Figure 1:**
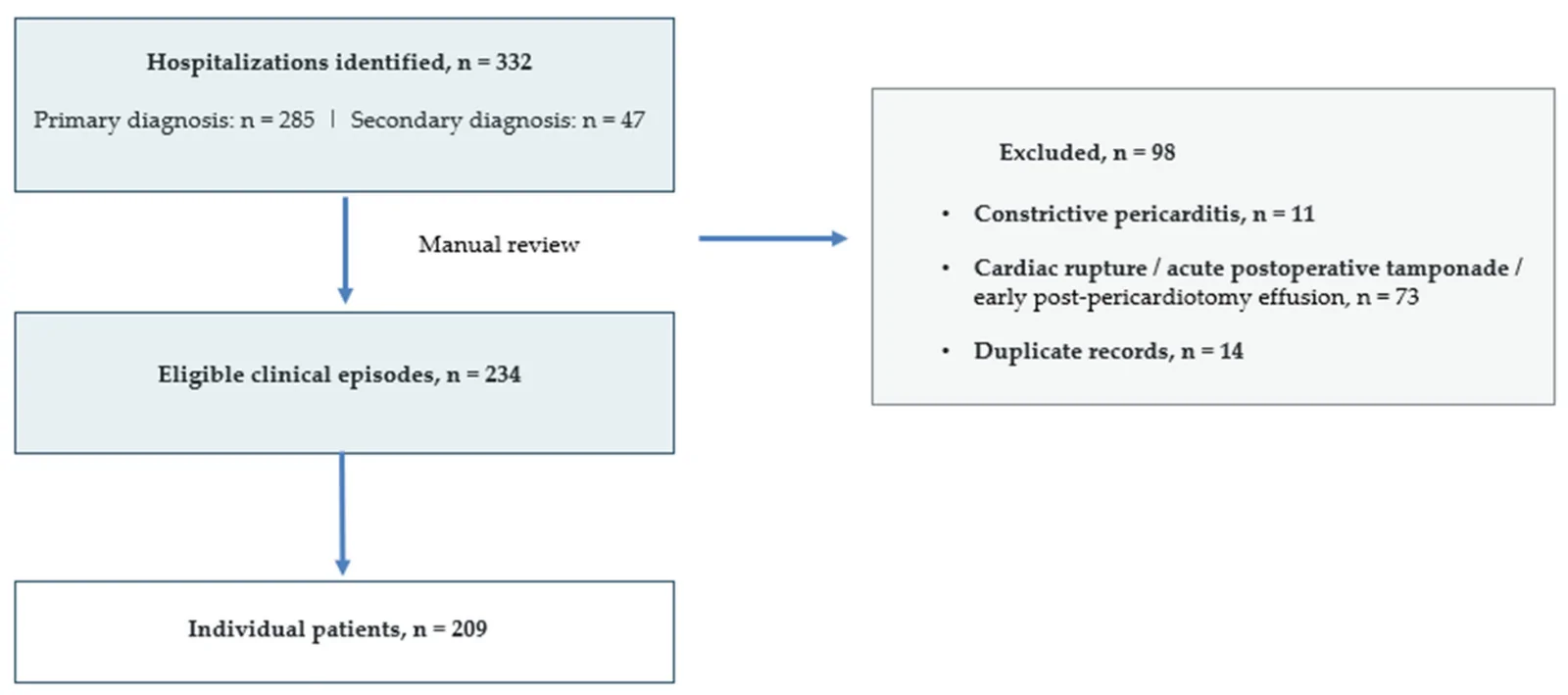
Flow diagram of study cohort selection.

After application of these criteria, the final study cohort comprised 234 eligible clinical episodes corresponding to 209 individual patients. Duplicate records referring to the same hospitalization were removed before construction of the final study database, whereas separate hospital admissions representing distinct clinical episodes were retained as individual cases. The cohort selection process is summarized in Figure 1.

Repeated admissions were retained when they represented distinct clinical episodes, often reflecting unsuccessful conservative treatment, recurrent effusion following pericardiocentesis, or recurrence after surgical drainage. In some cases, newly suspected or subsequently confirmed malignancy also influenced the decision to perform a pericardial window to reduce the risk of fluid reaccumulation.

Clinical data were extracted directly from the institutional electronic medical record system using a standardized data collection protocol. Demographic characteristics, cardiovascular risk factors, relevant medical history, previous oncological diagnoses, presenting symptoms, laboratory investigations, electrocardiographic findings, echocardiographic parameters, additional imaging examinations when available, therapeutic management, procedural details, and in-hospital outcomes were systematically recorded. Particular attention was paid to the characteristics of pericardial involvement, the severity of pericardial effusion, the presence of hemodynamic compromise, the occurrence of cardiac tamponade, and the treatment strategy adopted, including conservative medical management, pericardiocentesis, or surgical intervention. Pericardial effusion severity and hemodynamic compromise were recorded according to the assessments documented in the clinical and echocardiographic reports. Cardiac tamponade was classified according to the diagnosis recorded in the discharge summaries.

Pathological assessment included cytological examination of pericardial fluid and/or histopathological examination of pericardial tissue. Specimen submission was not systematic throughout the study period and depended on clinical circumstances, previous oncological history, and laboratory availability. During the COVID-19 pandemic, pathological investigations were more frequently limited, while specimens were not consistently submitted in patients with an established oncological diagnosis. In other cases, pathological results were unavailable because specimens were unsuitable for examination or no conclusive report could be retrieved. When surgical specimens were submitted, pericardial fluid and tissue were generally examined together, although the retrospective database did not consistently preserve separate findings for each modality. Accordingly, “pathological assessment” refers to the evaluation of available pericardial specimens, whereas “pericardial fluid cytology” is used when cytological findings were specifically documented. Neoplastic pericardial involvement was defined by the identification of malignant cells in pericardial fluid and/or neoplastic infiltration of pericardial tissue. Pathological findings were classified according to the final diagnostic reports issued by the institutional pathology department. Detailed information on specimen processing, cell-block preparation, immunocytochemistry, and standardized reporting criteria was not consistently available in the retrospective clinical records. For patients in whom malignancy was first identified following the pericardial presentation, the original pathology reports were individually reviewed to distinguish findings obtained from pericardial fluid cytology from those established by pericardial tissue histopathology.

The primary objective was to assess the contribution of pericardial fluid cytology to the diagnosis of malignant pericardial involvement, particularly in patients with no previously known malignancy. We hypothesized that, in a subset of patients presenting with pericardial effusion, cytological examination could provide the first pathological indication of an otherwise unrecognized malignancy. Secondary analyses examined whether malignant pericardial involvement was associated with a distinct clinicopathological profile, including differences in the macroscopic appearance of the pericardial fluid, and whether cytological detection of malignancy varied according to the underlying primary tumor. We also explored associations between malignant pericardial involvement and selected clinical and laboratory characteristics. Given the potential systemic consequences of hemodynamically significant pericardial effusion, the relationship between hemodynamic compromise and biochemical markers of organ dysfunction, particularly hepatic injury, was examined separately.

Statistical analyses were performed using MedCalc Statistical Software (version 24.0; MedCalc Software Ltd., Ostend, Belgium). Categorical variables are presented as frequencies and percentages, while continuous variables were expressed as means with standard deviations or medians with interquartile ranges, according to their distribution. Fisher’s exact test was used for comparisons of categorical variables in 2 × 2 tables, and the Fisher–Freeman–Halton test with Monte Carlo estimation was applied to larger contingency tables with sparse observations. Continuous variables were compared using the Mann–Whitney U or Kruskal–Wallis test, as appropriate. Associations were expressed as odds ratios (ORs) with 95% confidence intervals (CIs). All tests were two-sided, with *p* < 0.05 considered statistically significant. The Benjamini–Hochberg procedure was applied to control the false discovery rate in laboratory comparisons according to cardiac tamponade. Missing data were not imputed, and analyses were performed using available observations. Secondary comparisons were considered exploratory. No multiplicity adjustment was applied to other secondary comparisons; therefore, their nominal *p*-values were interpreted as descriptive evidence rather than confirmatory findings.

To assess the potential influence of repeated admissions, a sensitivity analysis was performed by restricting the dataset to the first eligible hospitalization per patient, identified chronologically by admission year and hospital record number.

The study protocol was approved by the Ethics Committee of the Institute of Cardiovascular Diseases Timișoara (Approval No. 8314/7 October 2025) and the Ethics Committee of the “Victor Babeș” University of Medicine and Pharmacy Timișoara (Approval No. 43/25/July 2025). The study was conducted in accordance with the principles of the Declaration of Helsinki. Given the retrospective observational design and the exclusive use of anonymized data obtained during routine clinical care, informed consent was waived in accordance with institutional regulations.

## 3. Results

### 3.1. Study Population and Clinical Characteristics

The final study cohort comprised 234 eligible clinical episodes occurring in 209 individual patients over the five-year study period. Most patients contributed a single hospitalization, whereas 23 patients (11.0%) had more than one eligible admission for distinct episodes of pericardial disease; 21 patients had two eligible admissions and two had three. The mean age was 60.8 ± 14.6 years, with a median of 63 years (interquartile range [IQR], 52–71) and an overall range of 18–93 years. The sex distribution across the analyzed episodes was nearly balanced, with 119 episodes (50.9%) occurring in women and 115 (49.1%) in men. A previously established malignancy was documented in 96 of 234 episodes (41.0%), whereas 138 (59.0%) occurred in patients without a known malignancy at presentation. Bronchopulmonary malignancies constituted the predominant oncological category, followed by breast malignancies, while the remaining cases encompassed a heterogeneous spectrum of primary tumor sites.

Large pericardial effusions predominated, accounting for 213 of 234 episodes (91.0%); moderate effusions were documented in 20 (8.5%), and a small effusion in one (0.4%). Signs of hemodynamic compromise were present in 174 episodes (74.4%). Among the 201 episodes with available information on concomitant pleural involvement, pleural effusion was documented in 111 (55.2%). Dyspnea was the most common presenting symptom, occurring in 171 episodes (73.1%), followed by fatigue in 87 (37.2%) and orthopnea in 35 (15.0%). Chest or precordial pain was less common and was documented in 21 episodes (9.0%).

Baseline laboratory data were available for most episodes and are further analyzed according to the presence of cardiac tamponade in Section 3.7.

A sensitivity analysis restricted to the first eligible hospitalization per patient included 209 individuals. Pathological assessment was available in 85 cases, of which 19 (22.4%) demonstrated neoplastic involvement. This proportion was comparable to that observed in the primary episode-based analysis (21/95, 22.1%). Among patients with available pathological assessment, neoplastic findings were identified in 11 of 31 patients (35.5%) with previously known malignancy and 8 of 54 patients (14.8%) without a previous oncological diagnosis (OR, 3.16; 95% CI, 1.11–9.05).

### 3.2. Procedural Management and Early Postprocedural Course

**Figure 2:**
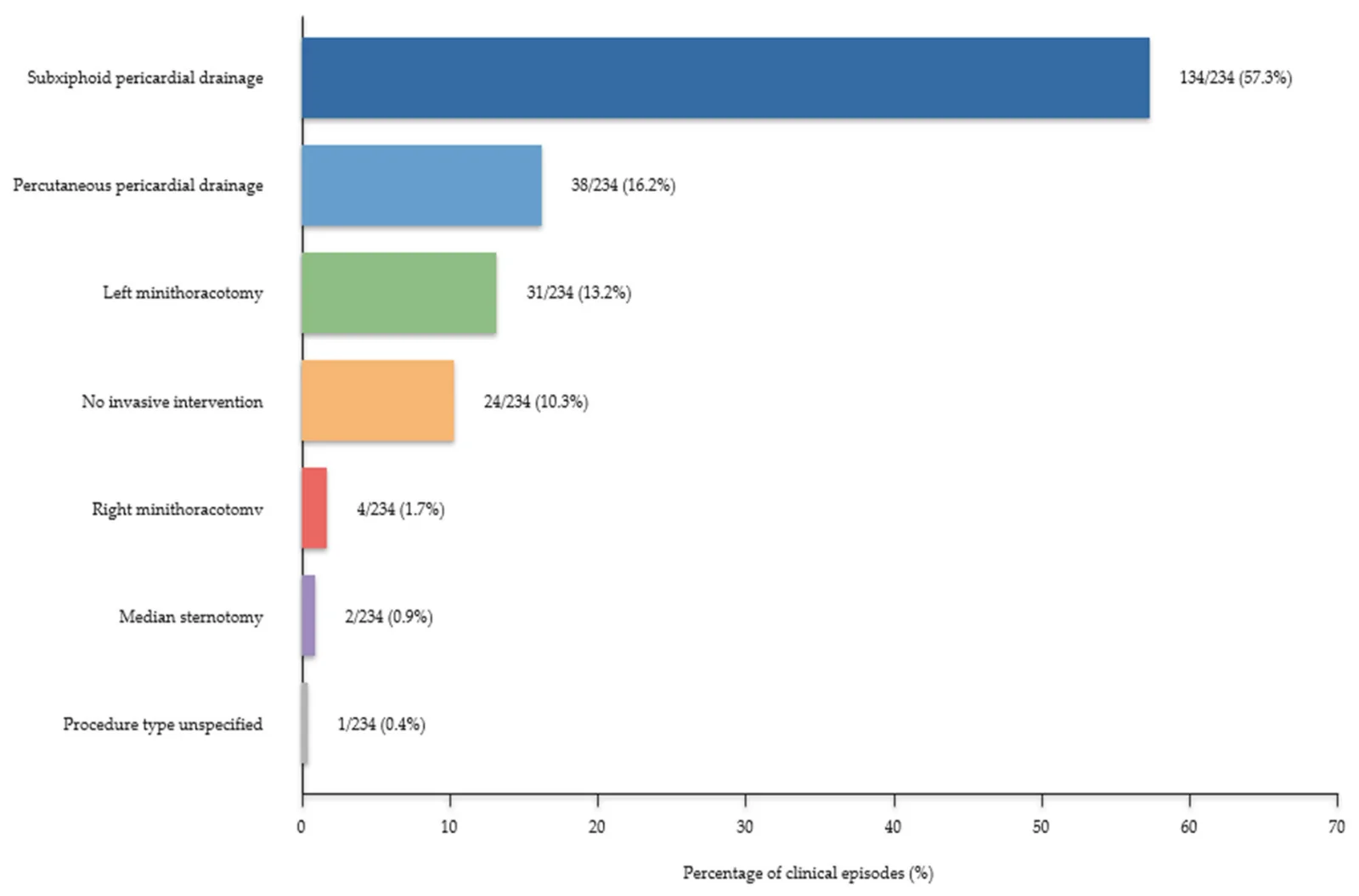
Distribution of procedural management strategies in the study cohort (*N* = 234 clinical episodes).

An invasive pericardial procedure was performed in 210 of the 234 clinical episodes (89.7%), whereas 24 episodes (10.3%) were managed without invasive intervention. Surgical treatment predominated, with subxiphoid pericardial drainage performed in 134 episodes (57.3% of the entire cohort), left minithoracotomy in 31 (13.2%), right minithoracotomy in four (1.7%), and median sternotomy in two (0.9%). Percutaneous pericardial drainage, including procedures documented as pericardiocentesis, was performed in 38 episodes (16.2%). The distribution of procedural management strategies is shown in Figure 2.

Cardiac tamponade was present in 140 of 171 surgically treated episodes (81.9%) and in 32 of 38 episodes managed by percutaneous drainage (84.2%). The frequency of tamponade did not differ significantly between the two procedural groups (Fisher’s exact *p* = 0.819). Drainage volume was documented in 207 episodes, with a median volume of evacuated pericardial fluid of 850 mL (IQR, 600–1100; range, 90–3700 mL).

Postprocedural mechanical ventilation was required in 151 surgically treated patients. Among those requiring intubation, the median duration of mechanical ventilation was 6 h (IQR, 4–10). All patients undergoing minithoracotomy or median sternotomy for whom intubation data were available required postoperative mechanical ventilation, while patients who did not require intubation within the surgical group had undergone subxiphoid drainage. Mechanical ventilation was uncommon following percutaneous drainage, being required in only three patients. Given the small number of intubated patients in this group, ventilation duration was not formally compared between surgical and percutaneous management. The median length of hospital stay for the overall cohort was 5 days (IQR, 4–7; range, 1–20 days).

Postoperative ventilation duration was also examined in relation to the primary malignancy among surgically treated patients with a previously established oncological diagnosis. Patients with pulmonary malignancies had a median ventilation duration of 6 h (IQR, 5–10.25) compared with 7 h (IQR, 5–12) in patients with other primary malignancies. This difference was not statistically significant (Mann–Whitney U, *p* = 0.505).

### 3.3. Pathological Evidence of Malignant Pericardial Involvement

Pathological assessment of pericardial specimens was available in 95 of the 234 clinical episodes (40.6%). Neoplastic involvement was identified in 21 of the 95 evaluable specimens (22.1%), while 74 (77.9%) showed no evidence of malignancy. Inflammatory findings accounted for 72 of the 74 non-neoplastic specimens (97.3%), with only two specimens showing other non-malignant findings.

**Figure 3:**
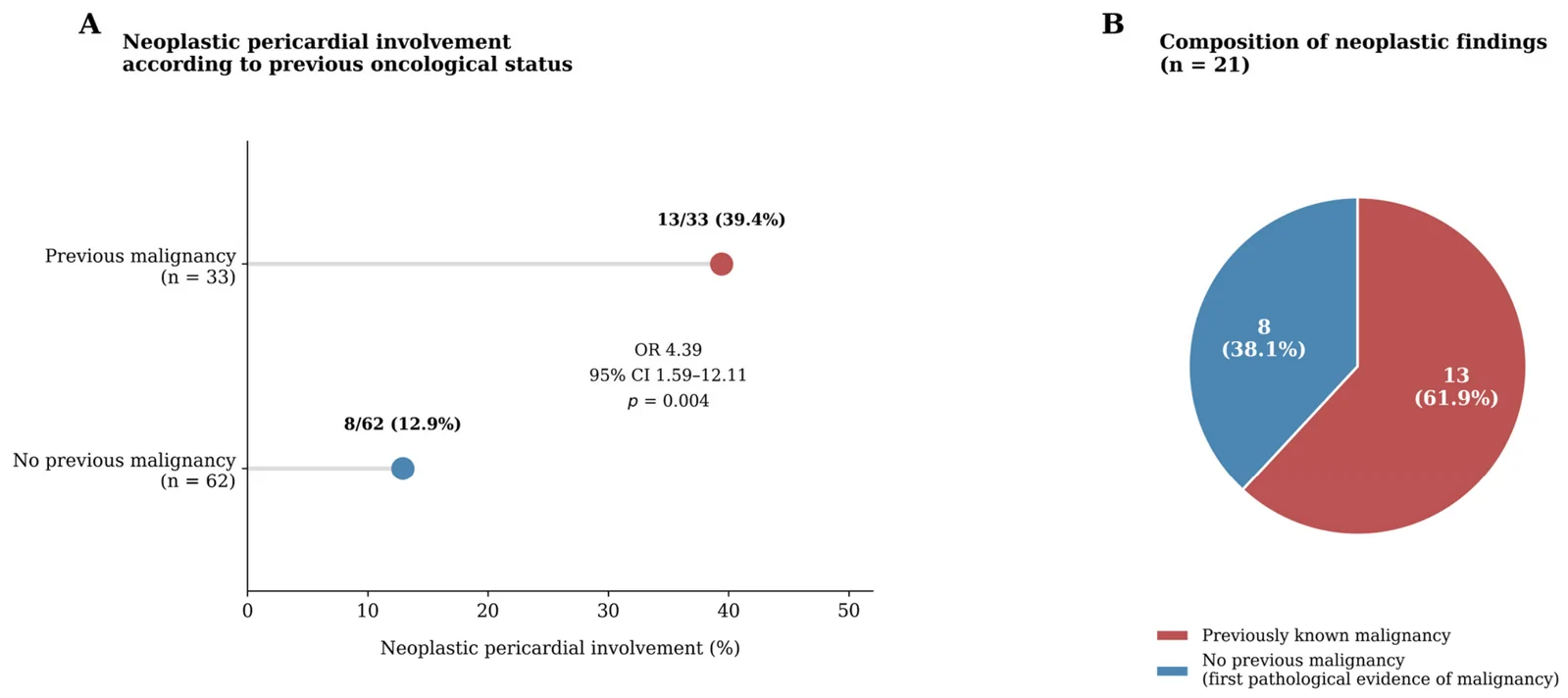
Neoplastic pericardial involvement according to previous oncological status. (**A**) Frequency of neoplastic involvement according to previous oncological status (OR 4.39, 95% CI 1.59–12.11; *p* = 0.004). (**B**) Distribution of neoplastic findings according to previous oncological status.

The frequency of neoplastic findings differed according to previous oncological status. Among the 33 evaluable episodes in patients with a previously established malignancy, neoplastic involvement was identified in 13 (39.4%). Among the 62 evaluable episodes in patients without a known malignancy, neoplastic findings were identified in 8 (12.9%), corresponding to an absolute difference of 26.5 percentage points. A previously established malignancy was associated with a more than fourfold higher odds of detecting neoplastic involvement in the examined pericardial specimens (odds ratio [OR], 4.39; 95% confidence interval [CI], 1.59–12.11; *p* = 0.004). The frequency and distribution of neoplastic pericardial involvement according to previous oncological status are shown in Figure 3.

Of the 21 episodes with neoplastic findings, 13 (61.9%) occurred in patients with a known malignancy and 8 (38.1%) in patients with no previously documented malignancy. Among patients with a previously established malignancy, 20 of the 33 evaluable episodes (60.6%) showed no evidence of neoplastic involvement in the examined specimens. Therefore, although a previous oncological diagnosis was associated with a higher frequency of neoplastic findings, malignant pericardial involvement was also documented in patients without a known malignancy.

The eight neoplastic findings identified in patients without a previously established malignancy represented 38.1% of all neoplastic pericardial findings and 12.9% of the evaluable episodes in patients without a known oncological diagnosis. In these cases, pericardial disease preceded the diagnosis of the underlying malignancy.

### 3.4. Diagnostic Contribution of Pericardial Fluid Cytology in Occult Malignancy

Positive pericardial fluid cytology was observed in two distinct clinical settings. In patients with a previously established malignancy, cytological examination confirmed malignant pericardial involvement in the context of an already recognized neoplastic disease. In eight patients, no malignancy had been documented before the pericardial presentation. In this subgroup, malignant cells identified in the pericardial fluid represented the first pathological evidence of an underlying neoplastic process.

The eight patients with previously unrecognized malignancy had a median age of 68 years (IQR, 65.5–72.5), and seven (87.5%) were women. Dyspnea was reported in all eight patients. Echocardiographic findings consistent with cardiac tamponade were documented in all cases, irrespective of pericardial effusion size. Orthopnea was documented in three patients (37.5%), while cough and fatigue were each present in three (37.5%). Concomitant pleural effusion was identified in four of the seven patients for whom this information was available (57.1%).

The pericardial fluid was hemorrhagic in three patients (37.5%), serosanguineous in three (37.5%), and clear yellow in two (25.0%). Thus, six of the eight patients (75.0%) had a hemorrhagic or blood-tinged effusion. The median volume of drained pericardial fluid was 750 mL (IQR, 550–1000; range, 90–1100 mL). Subxiphoid pericardial drainage was performed in five patients (62.5%), while three (37.5%) underwent percutaneous pericardial drainage.

Available pathological findings suggested a pulmonary origin in six of the eight patients (75.0%), including cases described as pulmonary adenocarcinoma, non-small-cell carcinoma, or pulmonary neoplasm. In Case 4, TTF-1 positivity was documented in the pericardial fluid cell sediment, while in Case 8, tissue immunohistochemistry demonstrated variable positivity for TTF-1 and Napsin A, supporting a pulmonary origin. In the remaining cases, the presumed pulmonary origin was based on the diagnostic interpretation recorded in the original pathology reports, without additional immunostaining results documented in the available records. One patient had an undifferentiated adenocarcinoma of undetermined origin, while another showed pathological findings suggestive of a possible breast primary. Therefore, pulmonary malignancies represented the predominant presumed primary category, although definitive confirmation of tumor origin was not available for all patients.

As pathological results were not always available before hospital discharge, subsequent diagnostic evaluation was coordinated by the treating physicians through referral to specialized pulmonary or oncology services. Investigations performed at other institutions were not included in the present retrospective dataset. The individual clinical characteristics, procedural approaches, and pathological findings of these eight patients are summarized in Table 1.

In Case 6, pericardial fluid cytology was the first pathological examination to identify malignant cells. Subsequent histopathological examination revealed intravascular tumor emboli and neoplastic infiltration, with strong CK7 and GATA3 positivity and negative TTF-1 staining. Despite the relatively small volume of pericardial fluid drained (90 mL), echocardiographic findings were consistent with cardiac tamponade, emphasizing that its hemodynamic significance is not determined by fluid volume alone.

**Figure 4:**
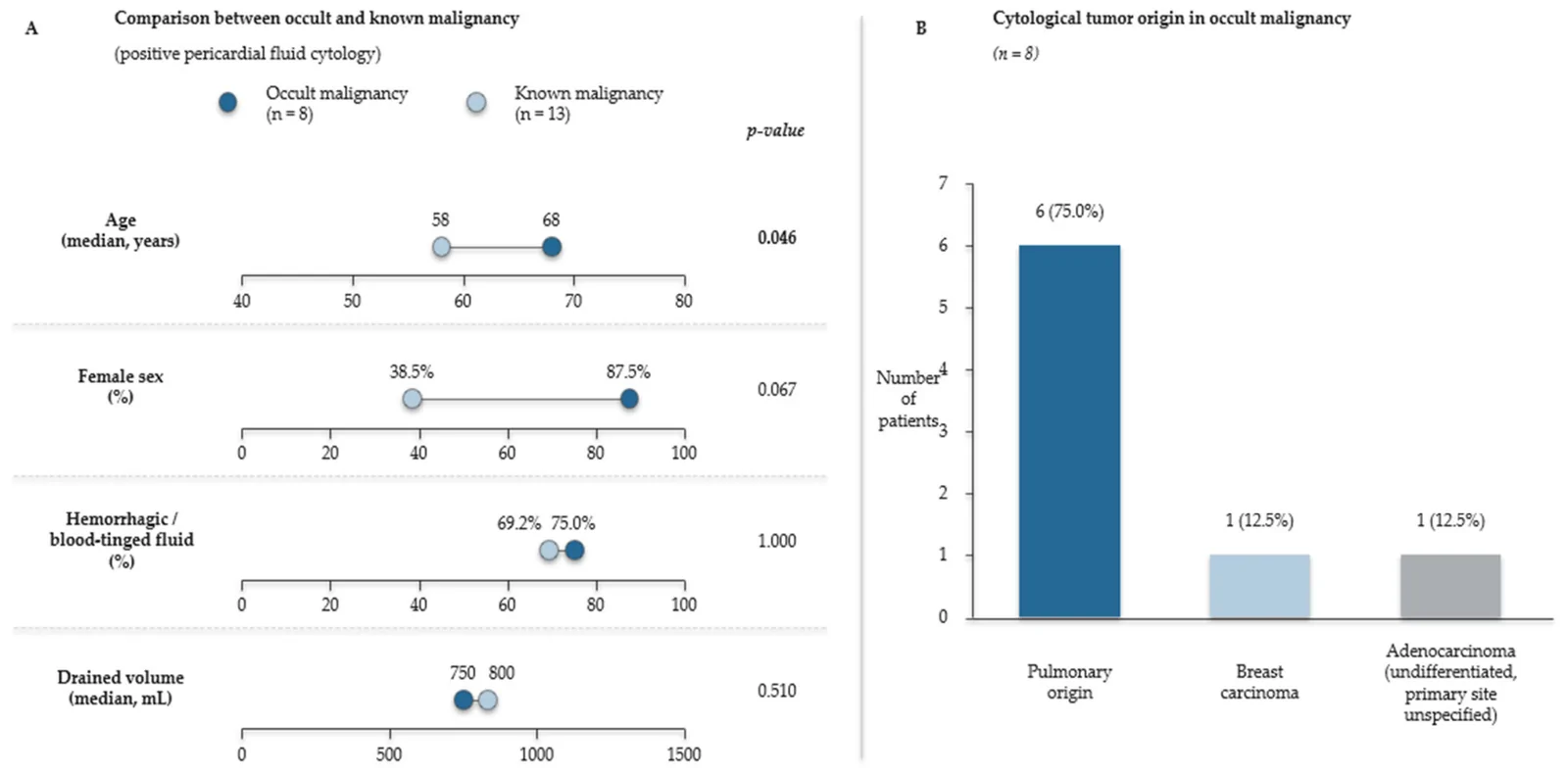
Characteristics and cytological tumor origin in patients with neoplastic pericardial involvement according to previous oncological status. (**A**) Comparative representation of demographic and clinical characteristics between patients with occult and previously known malignancy. (**B**) Distribution of cytologically identified tumor origins among patients with occult malignancy.

Patients with occult malignancy and positive pericardial fluid cytology were older than those with positive cytology and a previously established malignancy (median age, 68 vs. 58 years; *p* = 0.046). Women accounted for 87.5% and 38.5% of the two groups, respectively, although this difference did not reach statistical significance (*p* = 0.067). Hemorrhagic or blood-tinged pericardial fluid was similarly common in both groups (75.0% vs. 69.2%; *p* = 1.000), and the volume of drained fluid did not differ significantly (median, 750 vs. 800 mL; *p* = 0.510). No significant between-group differences were identified in hemoglobin (Hb), white blood cell (WBC) count, neutrophil proportion, erythrocyte sedimentation rate (ESR), alanine aminotransferase (ALT), aspartate aminotransferase (AST), serum creatinine, or length of hospital stay. The main characteristics and cytological tumor origin of patients with neoplastic pericardial involvement according to previous oncological status are summarized in Figure 4.

Pericardial fluid cytology provided the first pathological evidence of malignancy in all eight patients without a previously established oncological diagnosis. In each case, malignant cytological findings were obtained during the evaluation of the presenting pericardial effusion and preceded recognition of the underlying malignancy. Individual review of the original pathology reports confirmed the presence of malignant cells in pericardial fluid cytology in all eight patients.

### 3.5. Primary Tumor Type and Pericardial Cytological Findings

Pulmonary and bronchopulmonary malignancies were the most frequently represented tumors among patients with a previously established oncological diagnosis, accounting for 49 of 96 episodes (51.0%). Breast malignancies represented the second largest group (15/96, 15.6%), while the remaining primary tumor sites were individually less common. Pericardial fluid cytology was available in 33 of the 96 episodes (34.4%). The proportion of episodes with available cytological assessment was similar in patients with pulmonary and non-pulmonary malignancies (36.7% vs. 31.9%; *p* = 0.671).

Among episodes with available cytology, malignant cells were identified in 8 of 18 cases (44.4%) associated with pulmonary or bronchopulmonary malignancies and in 5 of 15 cases (33.3%) associated with non-pulmonary tumors. The absolute difference in cytological positivity was 11.1 percentage points (95% confidence interval [CI], −20.8 to 39.5). The corresponding odds ratio (OR) was 1.60 (95% CI, 0.39–6.62; Fisher’s exact *p* = 0.722).

The association between primary tumor type and cytological positivity was also assessed across the broader oncological spectrum. Tumors were grouped as pulmonary, breast, gastrointestinal, genitourinary/gynecological, hematological, and other malignancies. Several categories contained only a small number of patients with available cytology; therefore, the overall comparison was performed using the Fisher–Freeman–Halton exact test, with Monte Carlo estimation of the exact probability. No significant difference in the frequency of malignant cytology was identified across primary tumor categories (*p* = 0.182).

Erythrocyte sedimentation rate (ESR) was examined according to tumor origin as an exploratory measure of systemic inflammatory involvement. Median ESR was 32 mm/h (IQR, 25–45) in patients with pulmonary or bronchopulmonary malignancies and 50 mm/h (IQR, 17–100) in those with non-pulmonary tumors (*p* = 0.224). When ESR was compared across the broader primary tumor categories, no significant difference was observed (Kruskal–Wallis *p* = 0.564).

### 3.6. Clinicopathological Features Associated with Malignant Pericardial Involvement

The macroscopic appearance of the pericardial fluid differed numerically between episodes with and without neoplastic involvement. Neoplastic findings were identified in 6 of 44 episodes (13.6%) with clear-yellow fluid, 8 of 30 (26.7%) with serosanguineous fluid, and 7 of 20 (35.0%) with hemorrhagic fluid. When hemorrhagic and serosanguineous effusions were considered together, this appearance was documented in 15 of 21 episodes (71.4%) with neoplastic findings and in 35 of 74 (47.3%) without neoplastic involvement. The difference did not reach statistical significance (OR, 2.79; 95% CI, 0.97–7.97; Fisher’s exact *p* = 0.082).

Fluid appearance was also examined in relation to tumor origin. Among patients with a previously established malignancy and documented macroscopic appearance of the pericardial fluid, hemorrhagic or serosanguineous effusions were recorded in 33 of 45 episodes (73.3%) associated with pulmonary or bronchopulmonary malignancies, compared with 25 of 44 (56.8%) associated with other primary tumors. Although this appearance was more common in the pulmonary group, the difference was not statistically significant (OR, 2.09; 95% CI, 0.86–5.09; Fisher’s exact *p* = 0.123).

**Figure 5:**
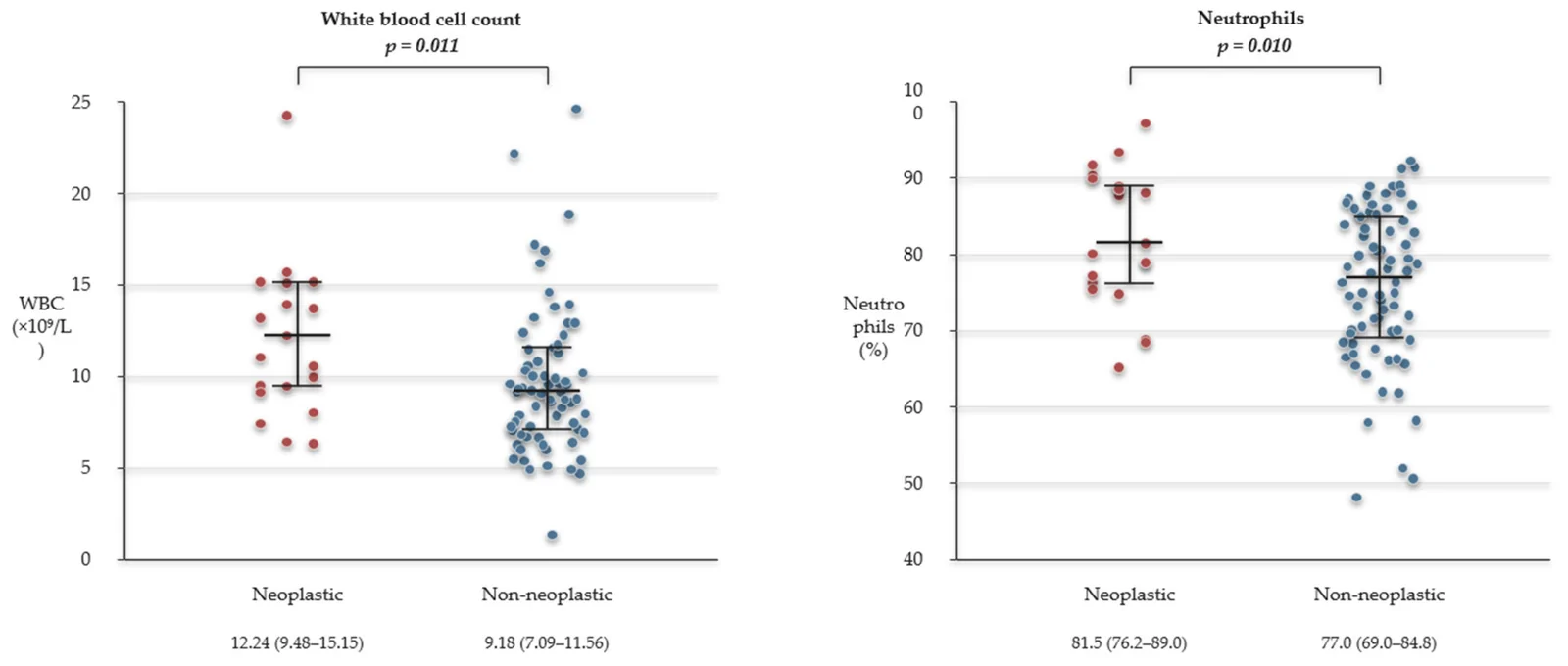
Laboratory findings according to neoplastic pericardial involvement. Dots represent individual observations from the study database; horizontal lines indicate medians and vertical bars indicate the interquartile range. Summary values and *p*-values correspond to those reported in the manuscript.

Differences were observed in the leukocyte profile. Episodes with neoplastic involvement had a higher white blood cell (WBC) count than those with non-neoplastic findings (median, 12.24 × 10^9^/L [IQR, 9.48–15.15] vs. 9.18 × 10^9^/L [IQR, 7.09–11.56]; *p* = 0.011). The neutrophil proportion was also higher in the neoplastic group (median, 81.5% [IQR, 76.2–89.0] vs. 77.0% [IQR, 69.0–84.8]; *p* = 0.010). In contrast, hemoglobin levels were comparable between groups (11.5 g/dL [IQR, 9.8–12.8] vs. 11.7 g/dL [IQR, 10.3–13.3]; *p* = 0.513), as were erythrocyte sedimentation rate (ESR) values (38 mm/h [IQR, 20–75] vs. 36 mm/h [IQR, 20–58]; *p* = 0.740). These laboratory findings according to neoplastic pericardial involvement are illustrated in Figure 5.

Hemodynamic compromise was documented in 20 of 21 episodes (95.2%) with neoplastic involvement and in 58 of 74 (78.4%) with non-neoplastic findings. Despite the numerical difference, the association did not reach statistical significance (OR, 5.52; 95% CI, 0.69–44.31; Fisher’s exact *p* = 0.107). Pleural effusion was similarly common in the two groups, occurring in 68.4% of episodes with neoplastic involvement and 64.5% of those without neoplastic findings (*p* = 1.000). The median volume of drained pericardial fluid was also similar (800 mL [IQR, 600–1000] vs. 900 mL [IQR, 600–1200]; *p* = 0.270). No significant differences were observed in age or sex distribution.

### 3.7. Laboratory Differences Associated with Cardiac Tamponade

Laboratory findings were compared between episodes with and without cardiac tamponade. Several differences were identified across hepatic, hematological, and leukocyte parameters, whereas renal function and several other routinely measured laboratory variables were comparable between the two groups. The complete analysis is presented in Table 2.

The largest differences were observed in serum transaminase levels. Both ALT and AST were higher in episodes presenting with cardiac tamponade, and both associations remained significant after adjustment for multiple comparisons (FDR-adjusted *p* < 0.001 for both). In contrast, serum creatinine did not differ between the two groups.

Cardiac tamponade was also associated with differences in the hematological profile. RBC count, Hb, and Hct were lower in episodes with tamponade, while WBC count and neutrophil proportion were higher and the lymphocyte proportion was lower. These differences remained significant after FDR correction. Platelet count and ESR were comparable between the two groups.

Among the remaining biochemical parameters, serum sodium was slightly lower in episodes with cardiac tamponade (median, 137 vs. 139 mmol/L), a difference that remained statistically significant after FDR adjustment (*p* = 0.016). Glucose and potassium levels did not differ significantly between groups. Although INR was higher in episodes with tamponade and remained significant after correction for multiple comparisons, this finding was not interpreted further because INR values may be influenced by anticoagulant therapy.

## 4. Discussion

### 4.1. Principal Findings and Diagnostic Role of Pericardial Cytology

Neoplastic pericardial involvement was identified in 21 of the 95 episodes with available pathological assessment (22.1%). Previous oncological status was strongly associated with this finding: malignant involvement was demonstrated in 13 of 33 evaluable episodes (39.4%) among patients with a known malignancy and in 8 of 62 (12.9%) among those without a previous oncological diagnosis (OR, 4.39; 95% CI, 1.59–12.11; *p* = 0.004). The proportion of malignant findings in our series can be placed within the range reported in large cytopathological cohorts, although differences in patient selection and specimen availability should be considered when comparing studies. Saab et al., in an analysis of 419 pericardial effusion specimens, identified malignant cytology in 62 specimens (14.8%) [18]. More recently, Takeda et al. reported malignancy in 99 of 309 cases (32.0%) in a multi-institutional cohort of patients with paired cytological and histological specimens [19], while Tannous et al. identified malignant cytology in 28 of 190 paired cases [20]. These series differ substantially in design and case selection, but collectively confirm that malignant disease represents an important component of the diagnostic spectrum encountered in patients undergoing pathological evaluation of pericardial effusion.

The association between a previous cancer diagnosis and malignant pericardial involvement in our cohort is consistent with the established pattern of secondary pericardial disease. Lung and breast cancers account for a substantial proportion of malignant pericardial effusions in published series [17]. In the systematic review by Shartouni et al., lung cancer, particularly non-small-cell lung cancer, was the most frequently reported primary malignancy, followed by breast, hematolymphoid, and gastrointestinal tumors [17]. This distribution is also reflected in contemporary ESC guidance, which describes metastatic pericardial involvement most commonly in association with lung cancer, breast cancer, lymphoma, and leukemia [5,7]. Pulmonary malignancy was likewise the most common pre-existing oncological diagnosis in our cohort, accounting for 49 of the 96 episodes occurring in patients with known cancer. The predominance of pulmonary malignancies in our population therefore follows the pattern reported internationally rather than representing a disease distribution specific to our center.

A previous diagnosis of cancer, however, did not reliably identify the etiology of the pericardial effusion. Of the 33 evaluable episodes in patients with established malignancy, 20 (60.6%) had no neoplastic involvement in the examined pericardial specimens. This observation is particularly relevant when interpreted alongside current guideline recommendations. The 2022 ESC Guidelines emphasize that pericardial effusion in a patient with cancer should not automatically be considered malignant and report that, in almost two-thirds of patients with documented malignancy, pericardial involvement may be related to non-malignant causes [5,7]. Definitive diagnosis of neoplastic pericardial disease requires demonstration of malignant infiltration by pericardial fluid cytology or pericardial biopsy [5]. The 2022 ESC Cardio-Oncology Guidelines similarly distinguish direct or metastatic pericardial involvement from treatment-related pericardial disease and other causes occurring in patients with cancer [5]. The 60.6% rate of non-neoplastic findings in our evaluable patients with known malignancy closely illustrates this clinical problem: oncological history increased the probability of malignant involvement more than fourfold, but pathological confirmation remained necessary to establish whether the pericardium was actually involved by malignancy.

This distinction also helps define the diagnostic value of pericardial fluid cytology. Several large series have shown that cytological examination performs at least comparably, and in some settings better, than pericardial biopsy for the detection of malignant involvement [17,18,19]. Saab et al. reported a sensitivity of 92.1% for cytology compared with 55.3% for pericardial biopsy [18]. In the more recent four-center study by Takeda et al., the corresponding sensitivities were 84.8% and 65.7% [19]. The systematic review by Shartouni et al. reached a similar conclusion, with cytology detecting malignant involvement more frequently than histology in most of the included comparative studies [17]. These observations are particularly pertinent to a clinical setting in which pericardial drainage is frequently undertaken for therapeutic reasons: analysis of the obtained fluid can simultaneously provide etiological information and determine whether malignant cells are present.

Our results also show the converse situation. Neoplastic involvement was identified in 12.9% of evaluable episodes occurring in patients without a previously known malignancy, and these eight cases represented 38.1% of all neoplastic findings in the study. Thus, pathological assessment operated in two different clinical directions within the same cohort. In patients with established cancer, it distinguished confirmed malignant involvement from pericardial disease without demonstrated neoplastic infiltration; in patients without a previous oncological diagnosis, it occasionally disclosed malignancy where it had not previously been recognized. This latter diagnostic pathway is particularly relevant to the central hypothesis of the present study and is examined separately in Section 4.2.

Current European guidance provides a relatively explicit framework for this approach. A similarly detailed contemporary AHA/ACC guideline specifically addressing malignant pericardial effusion and the diagnostic role of pericardial cytology is not currently available [2]. This absence should not be interpreted as disagreement with the European recommendations; rather, the degree of procedural and cytopathological detail devoted to malignant pericardial disease differs between currently available society guidance. Our findings are aligned with the ESC approach and underline the clinical value of establishing the etiology of a pericardial effusion rather than inferring it from oncological history alone [5].

### 4.2. Occult Malignancy Presenting as Pericardial Effusion

A particularly relevant finding of our study was the identification of eight patients in whom positive pericardial fluid cytology provided the first pathological evidence of malignancy. These patients had no previously established oncological diagnosis, yet all presented with a large pericardial effusion and hemodynamic compromise. Dyspnea was present in all eight patients, and six of the eight effusions (75%) were hemorrhagic or serosanguineous. The underlying malignancy was subsequently attributed to a pulmonary origin in six patients (75%), while one patient had breast cancer and one had an adenocarcinoma for which the primary site remained unspecified. Thus, in this subgroup, pericardial disease was not a complication occurring during the known course of cancer; it was the clinical event through which the malignant process first became pathologically apparent.

Although uncommon, this mode of presentation has been documented in larger pericardial cohorts. Ben-Horin et al. studied 173 consecutive patients undergoing pericardiocentesis for large, hemodynamically significant pericardial effusion. Neoplastic effusion was diagnosed in 58 patients, but cancer was already known in 45. In the remaining 13 patients, representing 7.5% of the entire cohort, the pericardial presentation disclosed a previously unrecognized malignancy; lung tumors predominated. When cases with an otherwise readily identifiable cause of the effusion were excluded, newly diagnosed cancer accounted for 18% of the remaining unexplained cases [13]. The clinical setting of that study is particularly relevant to our series because both populations were enriched in patients with clinically important effusions rather than incidentally detected small collections. Our eight cytology-positive cases similarly presented with large effusions accompanied by hemodynamic compromise, supporting the observation that an unexplained, clinically significant pericardial effusion may occasionally be the first manifestation of an underlying cancer.

A similar pattern was reported by Imazio et al. in a broader cohort of 450 patients with acute pericardial disease. A neoplastic etiology was identified in 33 patients (7.3%), and pericardial disease represented the first manifestation of a previously unknown malignancy in 18 patients (4.0% of the entire cohort). Lung cancer accounted for 72.2% of these newly recognized malignancies. Cardiac tamponade at presentation was associated with a substantially greater probability of a neoplastic etiology (OR, 7.0) [14]. The similarity with our subgroup is notable: six of our eight occult malignancies (75%) were of pulmonary origin, and all eight patients had hemodynamic compromise. The absolute proportions between studies should not be considered interchangeable because the populations and inclusion criteria differ, but the recurring association between severe pericardial presentation and an occult pulmonary malignancy is consistent across these independent cohorts.

The predominance of pulmonary malignancy in our occult group also agrees with older series specifically examining tamponade as the initial presentation of cancer. De la Gándara et al. described 13 patients in whom pericardial tamponade was the first manifestation of adenocarcinoma; the primary tumor could be established in ten, of whom seven had lung cancer. Pericardial fluid cytology established adenocarcinoma at the first examination in ten patients and after a second examination in two additional patients [23]. Earlier reports reached a similar conclusion. In a series of ten patients presenting with tamponade as the first manifestation of a cardiothoracic malignancy, El Allaf et al. documented the same uncommon but clinically important presentation [24], while Sulkes et al. specifically described malignant pericardial effusion leading to the diagnosis of previously unsuspected bronchogenic adenocarcinoma [25]. Although these older studies are smaller and should not carry the same evidentiary weight as the larger cohorts, they show that the association between an initial pericardial presentation and occult thoracic malignancy has been repeatedly observed over several decades.

The pulmonary predominance is also biologically plausible. The 2022 ESC Cardio-Oncology Guidelines describe neoplastic pericardial involvement as most commonly resulting from metastatic lymphatic spread, particularly from lung cancer, whereas hematogenous dissemination is emphasized for breast cancer [5]. Neoplastic involvement typically presents with a moderate-to-large effusion or cardiac tamponade rather than with isolated pericarditis, and definitive diagnosis rests on the demonstration of malignant infiltration by pericardial fluid cytology or pericardial biopsy. The 2022 ESC Cardio-Oncology Guidelines likewise recognize lung, esophageal, and breast cancers among tumors capable of direct pericardial involvement and note that malignancy-related effusions tend to develop gradually and may therefore become large before clinical recognition [5]. In our occult-malignancy subgroup, the combination of a large effusion, hemodynamic compromise, and a predominance of pulmonary primary tumors fits this established pattern of neoplastic pericardial disease.

The macroscopic appearance of the fluid deserves a more cautious interpretation. Six of the eight patients had blood-containing effusions, either hemorrhagic or serosanguineous. Hemorrhagic fluid has traditionally raised suspicion of malignancy, particularly in large unexplained effusions, but it is not specific for a neoplastic etiology and should not be used independently to classify an effusion as malignant [5,17]. In our study, its relevance lies instead in the combination of findings: a large effusion with hemodynamic consequences, blood-containing fluid, and positive cytology in a patient without a previous cancer diagnosis. This distinction is important because the cytological finding, rather than the gross appearance of the fluid, provided the pathological evidence of malignancy. The broader relationship between fluid appearance and neoplastic involvement in the complete pathological cohort is addressed separately in Section 4.3.

Our findings also illustrate why the diagnostic value of pericardial drainage extends beyond immediate hemodynamic relief. In the eight patients with occult malignancy, five underwent subxiphoid drainage and three underwent percutaneous drainage. In each case, the procedure provided material from which malignant cells were identified. This is particularly relevant in patients presenting to cardiovascular or surgical services before an oncological diagnosis has been established: the indication for drainage may initially be driven by the size of the effusion and its hemodynamic effect, while cytological examination of the retrieved fluid may alter the subsequent diagnostic trajectory. The ESC/ACVC consensus on acute pericardial disease in cancer similarly recognizes the importance of etiological investigation of pericardial effusion and includes cytological examination among the analyses of pericardial fluid obtained during drainage [5,26].

The present series therefore adds a contemporary surgical cohort to an observation that has been described previously but remains clinically important: an apparently primary pericardial presentation may occasionally represent the first detectable expression of an extracardiac malignancy. The close agreement between the pulmonary predominance observed in our patients (6/8, 75%) and that reported by Imazio et al. among previously undiagnosed cancers (72.2%) is particularly noteworthy [14]. More importantly, the diagnostic sequence in our patients was consistent: clinically significant pericardial disease prompted drainage, cytological examination demonstrated malignant cells, and this finding provided the first pathological evidence of the underlying cancer. Therefore, in patients presenting with a large and otherwise unexplained pericardial effusion, particularly when accompanied by hemodynamic compromise, pericardial fluid should be regarded not only as therapeutic drainage material but also as a potentially decisive diagnostic specimen [5].

### 4.3. Clinical, Laboratory, and Procedural Implications

Beyond cytological diagnosis, several clinical and laboratory findings in our cohort helped define the phenotype associated with neoplastic pericardial involvement. Blood-containing fluid was more common among neoplastic than non-neoplastic specimens (71.4% vs. 47.3%), corresponding to an OR of 2.79, although the association did not reach statistical significance (*p* = 0.082). The gradient across individual macroscopic categories followed the same direction: neoplastic involvement was identified in 13.6% of clear-yellow, 26.7% of serosanguineous, and 35.0% of hemorrhagic effusions. These findings are consistent with the long-recognized association between malignant pericardial disease and hemorrhagic effusion [17], but they also show why gross appearance cannot be used as a surrogate for pathological diagnosis. Almost half of the non-neoplastic specimens in our series were obtained from blood-containing effusions, while malignant cells were also found in clear-yellow fluid. The macroscopic appearance of the effusion may therefore raise suspicion, but cytological or histological confirmation remains necessary [5].

The inflammatory profile provided an additional signal. Patients with neoplastic involvement had higher peripheral leukocyte counts than those with non-neoplastic findings [median 12.24 vs. 9.18 × 10^9^/L; *p* = 0.011] and a higher neutrophil percentage [81.5% vs. 77.0%; *p* = 0.010], whereas hemoglobin and erythrocyte sedimentation rate did not differ significantly between the groups. These differences are best interpreted as features accompanying malignant pericardial disease rather than as diagnostic markers. Current ESC guidance does not identify peripheral leukocytosis or neutrophilia as criteria for diagnosing neoplastic pericardial involvement; definitive diagnosis remains dependent on malignant cells in pericardial fluid or malignant infiltration on biopsy [5]. The discordance between leukocyte/neutrophil counts and ESR in our cohort is also relevant: it suggests that the observed systemic inflammatory response was not captured uniformly by conventional inflammatory indices. From a clinical standpoint, neither leukocytosis nor neutrophilia appears sufficiently specific to replace direct pathological assessment.

Hemodynamic compromise was common throughout the study population and particularly common among patients with neoplastic findings. It was present in 20 of 21 neoplastic episodes (95.2%), compared with 58 of 74 non-neoplastic episodes (78.4%). The estimated OR was 5.52, but with a wide confidence interval (95% CI, 0.69–44.31) and without statistical significance (*p* = 0.107). The direction of this association is nevertheless consistent with the recognized clinical presentation of neoplastic pericardial disease [11]. The 2022 ESC Guidelines describe malignant pericardial involvement as more commonly presenting with a moderate-to-large effusion or cardiac tamponade than with isolated pericarditis and list malignancy among the most common causes of tamponade [5]. Our results add an important clinical qualification: hemodynamic compromise was also common in the non-neoplastic group. Its presence should therefore trigger prompt treatment and etiological investigation, but does not identify malignancy by itself.

The systemic consequences of hemodynamic impairment were also apparent in the laboratory analysis of the complete cohort. Episodes with tamponade were associated with significantly higher alanine aminotransferase (ALT) and aspartate aminotransferase (AST) levels (both *p* < 0.001 after false discovery rate correction), together with differences in hemoglobin, hematocrit, erythrocyte count, sodium, leukocyte count, neutrophil percentage, and lymphocyte percentage. Creatinine, platelet count, and ESR were not significantly different. These findings are compatible with the systemic effects of impaired cardiac filling and reduced forward flow during tamponade rather than with a tumor-specific laboratory pattern. The ESC defines cardiac tamponade as the point at which pericardial pressure impairs diastolic filling sufficiently to reduce cardiac output; importantly, the hemodynamic consequences depend not only on effusion volume but also on the rate of accumulation and pericardial compliance [5,8,9]. The marked transaminase differences observed in our cohort are therefore physiologically plausible in the setting of systemic venous congestion and/or reduced hepatic perfusion. They may be useful as markers of the systemic impact of tamponade, but not as indicators of the underlying pericardial etiology.

The procedural profile of our cohort reflects this high burden of clinically significant effusion. An invasive drainage procedure was required in 210 of 234 episodes (89.7%). Surgical drainage predominated: 134 patients underwent a subxiphoid procedure, 31 underwent a left minithoracotomy, four underwent a right minithoracotomy, and two underwent a sternotomy, whereas percutaneous drainage was performed in 38 episodes. The median drained volume was 850 mL (IQR, 600–1100 mL). Despite the different procedural approaches, the frequency of tamponade was similar among patients treated surgically and those managed percutaneously (81.9% vs. 84.2%; *p* = 0.819). This is important when interpreting procedural choice in our series: the presence of tamponade alone did not distinguish patients selected for surgery from those undergoing percutaneous drainage.

Current guidance supports drainage as the definitive treatment for cardiac tamponade and recommends image-guided pericardiocentesis without delay in unstable patients [5]. Surgery remains appropriate when percutaneous drainage is not feasible and in selected situations requiring more complete drainage or creation of a pericardial window [5]. For recurrent effusions with hemodynamic compromise, the ESC specifically recognizes surgical pericardiotomy or a pleuropericardial window as therapeutic options. In neoplastic effusions, extended drainage may be necessary because recurrence exceeds 50%, and pericardiotomy, surgical window, or balloon pericardiotomy can be considered for recurrent disease [27,28,29,30,31,32]. This is particularly relevant to a cardiovascular surgical cohort such as ours, in which drainage is not merely an emergency maneuver but may also provide durable decompression and access to pathological material.

Comparative evidence also helps place our procedural experience in context. A 2025 systematic review and meta-analysis including 12 studies and 3721 patients with malignant pericardial effusion found a higher risk of effusion re-accumulation after pericardiocentesis than after surgical pericardiotomy (OR, 3.81; 95% CI, 1.99–7.31), while in-hospital mortality, bleeding, atrial fibrillation, hemodynamic instability, and length of stay did not differ significantly between strategies [29]. These data do not establish a universally preferable procedure; rather, they underline that the objectives of intervention differ between patients. Rapid decompression may be the immediate priority in unstable tamponade, whereas recurrent effusion, anatomical characteristics, need for tissue sampling, overall oncological status, and expected prognosis may influence the decision to use a surgical approach [5,29].

This dual therapeutic and diagnostic role is particularly relevant to the interpretation of our cohort. Drainage relieved a potentially life-threatening hemodynamic condition, but it also generated the fluid—and, with surgical approaches, potentially tissue—required for etiological diagnosis. The 2022 ESC Guidelines explicitly indicate pericardiocentesis not only for tamponade and symptomatic moderate-to-large effusions but also when etiological investigation is required, including suspected malignancy [5]. Pericardial biopsy, when indicated, is commonly obtained during thoracoscopic or surgical drainage or at the time of a pericardial window [5]. In this setting, the procedure and the diagnostic work-up should not be regarded as separate steps. For patients with significant pericardial effusion, particularly when malignancy is among the possible causes, drainage provides an opportunity to address the hemodynamic problem while obtaining material capable of defining the underlying disease.

### 4.4. Strengths, Limitations, and Future Perspectives

Previous studies have examined malignant pericardial effusion from perspectives that differ from the specific question addressed in the present study. Large cytopathological series have primarily evaluated the diagnostic yield of pericardial fluid cytology and its concordance with pericardial biopsy. Saab et al., for example, analyzed 419 pericardial effusion specimens and directly compared cytological and biopsy findings in paired samples [18], while the recent multi-institutional study by Takeda et al. was specifically designed to compare the sensitivity of cytology and biopsy in malignant effusions [19]. Tannous et al. similarly examined cytology–histology correlation in 190 cases with both cytological evaluation and a pericardial surgical specimen [20]. These studies provide stronger evidence for the diagnostic performance of cytology itself, but they were not designed to address the same clinical question as our analysis. Our cohort was assembled from clinical episodes rather than from pathology records, allowing pathological findings to be interpreted in relation to the patient’s previous oncological status, clinical presentation, hemodynamic consequences, and procedural management. This distinction enabled us to examine two clinically different situations within the same cohort: confirmation of neoplastic pericardial involvement in patients with known cancer and detection of malignancy in patients with no previous oncological diagnosis.

The latter represents a particular strength of the present analysis. Earlier clinical cohorts established that significant pericardial disease can be the presenting manifestation of an otherwise unrecognized malignancy. Ben-Horin et al. identified previously unknown cancer in 13 of 173 patients undergoing pericardiocentesis for hemodynamically significant effusion [13], whereas Imazio et al. reported pericardial disease as the first manifestation of malignancy in 18 of 450 patients with acute pericardial disease [14]. Our study approaches this phenomenon from a more specific diagnostic perspective by identifying patients in whom pericardial fluid cytology itself provided the first pathological evidence of malignancy. Eight of 62 pathologically evaluated episodes in patients without a previous cancer diagnosis (12.9%) fulfilled this definition, accounting for 38.1% of all neoplastic findings in the pathological cohort. Conversely, only 13 of 33 evaluable episodes in patients with known malignancy showed neoplastic pericardial involvement. The ability to examine these two directions within the same clinical cohort highlights the contribution of pericardial pathological assessment beyond knowledge of the patient’s oncological history: it may confirm pericardial involvement in an established malignancy, but it may also uncover a malignant process that had not previously been diagnosed.

Several characteristics of the study design further support this clinical perspective. The cohort covered five consecutive years and included 234 clinical episodes in 209 patients, with case identification and eligibility independently reviewed by two investigators through three screening passes and disagreements resolved by consensus. The available data allowed pathological findings to be linked with symptoms, effusion size, hemodynamic compromise, pleural effusion, laboratory findings, macroscopic fluid appearance, drainage volume, and procedural approach. This level of clinical and procedural characterization differs from specimen-based cytopathological studies [18,19,20] and is particularly relevant to patients receiving cardiovascular or surgical services with clinically significant effusions. At the same time, this referral pattern defines the population to which our findings are most directly applicable. Large effusions and hemodynamic compromise were highly prevalent in our cohort, and the results should therefore not be extrapolated to the full spectrum of pericardial effusion encountered in general cardiology or asymptomatic populations. Furthermore, because cohort identification was restricted to admissions coded for pericarditis within the Department of Cardiovascular Surgery, patients assigned exclusively other diagnostic codes or admitted to other departments may not have been captured.

The retrospective, single-center design also imposes important constraints. Pathological assessment was available in 95 of the 234 episodes (40.6%) and was obtained according to clinical and procedural circumstances rather than through a study-mandated sampling protocol. Specimen submission was also influenced by previous oncological diagnoses and restrictions affecting diagnostic investigations during the COVID-19 pandemic. In some cases, pathological findings were unavailable because specimens were unsuitable for examination or no conclusive report could be retrieved. Furthermore, the retrospective database did not consistently preserve separate cytological and histopathological results, preventing a reliable assessment of the individual contribution of each modality. Consequently, the 22.1% proportion of neoplastic findings describes the pathologically evaluated subgroup and should not be interpreted as the prevalence of malignant pericardial effusion in the entire cohort. Cytological and histological specimens were not systematically paired, unlike the designs used by Takeda et al. and Tannous et al. [19,20]; therefore, our study was neither designed nor able to estimate the comparative sensitivity of the two methods or the diagnostic accuracy of pericardial cytology. These differences are methodological rather than contradictory: the previous studies establish the diagnostic performance of pericardial cytology, whereas our analysis examines its contribution within a defined clinical and oncological context. Because pathological assessment was not systematically performed, the evaluable subgroup may differ from the overall cohort in terms of clinical severity, oncological history, and therapeutic management. This potential selection bias limits the generalizability of the pathological findings, which should not be interpreted as reflecting the prevalence of malignant pericardial involvement in the entire cohort.

Pericardial fluid may also provide valuable material for molecular characterization, particularly in patients with suspected or confirmed pulmonary malignancy. However, the potential benefits of advanced molecular testing should be considered alongside the need to establish consistent access to basic cytological assessment. In our cohort, pathological evaluation was available in only 40.6% of episodes, highlighting an important gap in the initial diagnostic pathway. Improving specimen collection, processing, and access to cytological examination should therefore represent an immediate priority. Once malignant involvement is identified or clinically suspected, appropriately preserved and sufficiently cellular specimens may support additional biomarker testing, guided by oncological indications and available resources. Molecular analyses were not performed in the present study, and their feasibility and clinical utility in this setting require further investigation.

**Figure 6:**
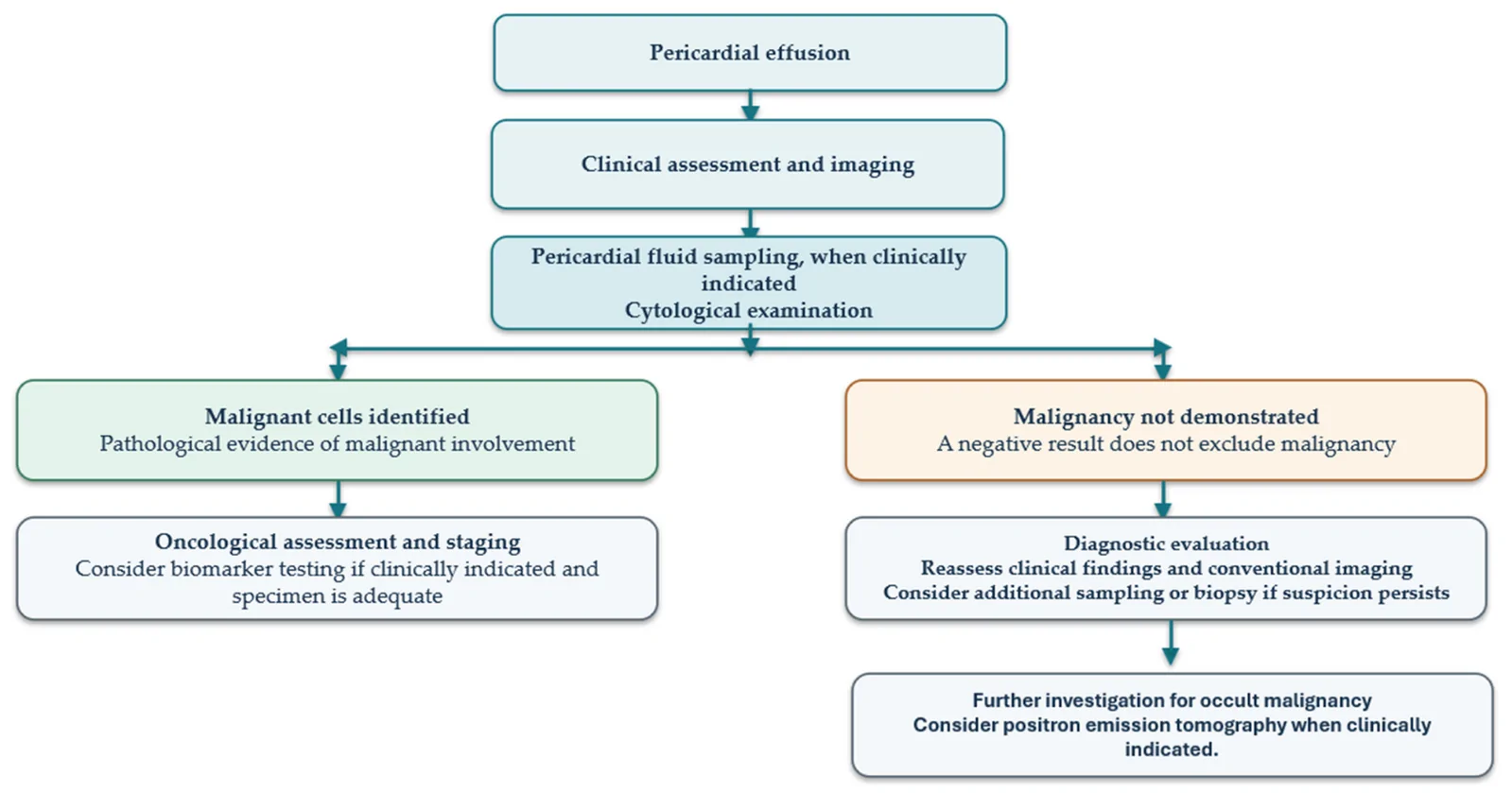
Proposed clinical pathway for suspected malignant pericardial involvement.

To illustrate the potential clinical implications of these findings, we propose a stepwise diagnostic pathway for patients with suspected malignant pericardial involvement (Figure 6). This approach emphasizes the integration of pericardial fluid cytology with clinical assessment and imaging, while recognizing that negative cytological findings do not exclude malignancy. Further investigations, including additional tissue sampling, advanced imaging, or molecular testing, may be considered according to clinical suspicion and specimen availability. Importantly, this proposed pathway is conceptual and was not prospectively evaluated or validated in the present study [18,19].

The number of pathologically confirmed neoplastic episodes was relatively small (*n* = 21), and the subgroup in which cytology disclosed a previously unknown malignancy comprised eight patients. Associations derived from these groups, particularly those involving fluid appearance, inflammatory parameters, or clinical phenotype, should therefore be regarded as exploratory rather than predictive. Given the multiple secondary comparisons and limited subgroup sizes, nominally significant findings, particularly those close to the conventional significance threshold, should be interpreted cautiously and considered hypothesis-generating. Some variables were incompletely documented, resulting in variable denominators across individual analyses. Although the primary analysis retained clinically distinct readmissions, the resulting within-patient dependence was addressed through a sensitivity analysis restricted to the first eligible episode per patient. The proportion of neoplastic pathological findings remained comparable to that observed in the episode-based analysis. Nevertheless, this approach does not capture subsequent changes in clinical presentation, oncological status, or therapeutic management. Finally, follow-up was limited to the index hospitalization. Subsequent cancer investigations, treatment, recurrence of pericardial effusion, repeat sampling, and long-term outcomes were therefore outside the scope of the present dataset. In particular, the absence of systematic longitudinal verification among patients with non-neoplastic pathological findings precludes estimation of the false-negative rate or negative predictive value of cytology in this cohort.

Future studies could combine the complementary strengths of clinical and cytopathological approaches. A prospective multicenter cohort of consecutive patients undergoing drainage for clinically significant pericardial effusion, with standardized cytological processing, histological sampling when clinically indicated, and longitudinal oncological follow-up, would allow the diagnostic pathway to be evaluated more completely. Standardized cytopathological reporting may also improve comparisons across institutions and provide a five-category framework for serous fluid specimens; a recent pericardial-specific meta-analysis including 2976 samples from ten studies demonstrated progressive stratification of malignancy risk across these categories while also identifying substantial between-study heterogeneity [33,34]. Prospective application of such standardized reporting could facilitate more reproducible assessment of pericardial cytology. Most importantly for the question raised by our findings, larger longitudinal cohorts are needed to determine which combination of clinical, imaging, hemodynamic, and cytological features should prompt systematic investigation for an occult malignancy in patients presenting with an otherwise unexplained, clinically significant pericardial effusion.

## 5. Conclusions

The present study demonstrates that the diagnostic contribution of pericardial fluid cytology extends beyond the conventional confirmation of malignant pericardial involvement. Previous oncological status was strongly associated with neoplastic findings, with a more than fourfold higher odds of pericardial involvement among patients with known malignancy. However, 60.6% of pathologically evaluated episodes in this group showed no evidence of neoplastic involvement. This finding is clinically relevant because it indicates that pericardial effusion in a patient with cancer should not be presumptively attributed to the underlying malignancy. Cytological assessment should therefore not be considered redundant in patients with an established oncological diagnosis as it may distinguish true neoplastic involvement from pericardial disease of another etiology.

More importantly, our primary hypothesis was supported by the identification of eight patients without a previous oncological diagnosis in whom pericardial fluid cytology provided the first pathological evidence of malignancy. These cases accounted for 12.9% of pathologically evaluated episodes among patients without known cancer and 38.1% of all neoplastic findings. All presented with large pericardial effusion and hemodynamic compromise, indicating that pericardial effusion or tamponade may act as a sentinel manifestation of an otherwise occult malignancy, initiating the subsequent oncological diagnostic pathway.

Cytology may also provide information beyond the binary distinction between malignant and non-malignant effusion. Six of the eight occult malignancies identified in our cohort were cytologically consistent with a pulmonary origin, including cases characterized as pulmonary adenocarcinoma or non-small-cell carcinoma. Although primary tumor type was not significantly associated with cytological positivity across the broader oncological cohort, these findings suggest that, when malignant cells are detected in a patient without known cancer, their cytomorphological and immunocytochemical characteristics may help orient the subsequent search for the primary tumor. Pericardial cytology may therefore contribute not only to malignancy detection, but also to tumor-origin attribution in selected patients.

The secondary analyses showed that blood-containing fluid, peripheral leukocytosis, neutrophilia, and hemodynamic compromise were more prominent in patients with neoplastic involvement, although none provided sufficient specificity to replace pathological assessment. Similarly, cardiac tamponade was associated with significant ALT and AST elevations and additional hematological changes, supporting the systemic consequences of severe hemodynamic impairment. These findings characterize the clinical setting in which malignant pericardial disease may occur but reinforce the central role of direct examination of the pericardial specimen.

The principal contribution of this study is therefore the identification of a bidirectional diagnostic role of pericardial cytology. In patients with established malignancy, it can determine whether a new pericardial presentation represents demonstrable neoplastic involvement rather than being attributed to cancer solely on clinical grounds. In patients without known malignancy, a large pericardial effusion or tamponade may constitute the first manifestation of cancer, with cytology providing both the first pathological evidence of malignancy and, in selected cases, an indication of its likely primary origin. This diagnostic pathway directly supports the central premise of our study—occult malignancy presenting as pericardial effusion—and argues for systematic cytological evaluation of pericardial fluid obtained during clinically indicated drainage, irrespective of whether malignancy is already known or only suspected.

## Figures and Tables

**Table 1:** Patient-level characteristics of eight cases in which pericardial cytology provided the first pathological evidence of malignancy.

Case	Age (years)	Sex	Pericardial Fluid Appearance	Drained Volume (mL)	Drainage Procedure	Pericardial Fluid Cytology	Pericardial Tissue Histopathology	Immunostaining Findings
1	79	F	Serosanguineous	600	Subxiphoid drainage	Malignant cells; findings consistent with pulmonary adenocarcinoma	No malignant involvement identified	Not performed
2	74	F	Clear yellow	400	Subxiphoid drainage	Malignant cells; findings consistent with pulmonary adenocarcinoma	No malignant involvement identified	Not performed
3	66	M	Hemorrhagic	1000	Pericardiocentesis	Malignant cells; undifferentiated adenocarcinoma	No malignant involvement identified	Not performed
4	49	F	Hemorrhagic	800	Subxiphoid drainage	Malignant cells; findings suggestive of pulmonary origin	No malignant involvement identified	TTF-1 positive (pericardial fluid cell sediment)
5	72	F	Clear yellow	1000	Pericardiocentesis	Malignant cells; findings consistent with pulmonary non-small-cell adenocarcinoma	No malignant involvement identified	Not performed
6	70	F	Bloody	90	Subxiphoid drainage	Malignant cells with marked nuclear atypia, pleomorphism, hyperchromasia, and mitotic activity	Intravascular tumor emboli and neoplastic infiltration; primary site not established morphologically	CK7 strongly positive; GATA3 strongly positive; TTF-1 negative (pericardial tissue)
7	64	F	Serosanguineous	1100	Pericardiocentesis	Malignant cells; findings consistent with pulmonary non-small-cell adenocarcinoma	No malignant involvement identified	Not performed
8	66	F	Hemorrhagic	700	Subxiphoid drainage	Malignant cells identified	Tumor cells identified; immunophenotype supporting pulmonary origin	TTF-1 and Napsin A variably positive; thyroglobulin, PAX8, and GATA3 negative; calretinin positive only in mesothelial cells (pericardial tissue)

Note: F, female; M, male; TTF-1, thyroid transcription factor 1; CK7, cytokeratin 7; GATA3, GATA-binding protein 3; PAX8, paired box gene 8. Cytological, histopathological, and immunostaining findings were verified against the original pathology reports. Immunostaining results are reported only when explicitly documented and are distinguished according to specimen type. Subsequent diagnostic investigations and final oncological diagnoses could not be systematically verified because records from external institutions were unavailable.

**Table 2:** Laboratory findings according to the presence of cardiac tamponade.

Laboratory Parameter	Cardiac Tamponade, Median (IQR), *n*	No Cardiac Tamponade, Median (IQR), *n*	*p*-Value	FDR-Adjusted *p*-Value
ALT, U/L	44 (23–95), 173	25 (18–41), 59	**<0.001**	**<0.001**
AST, U/L	34 (22–83), 173	22 (17–34), 59	**<0.001**	**<0.001**
Creatinine, mg/dL	1.08 (0.83–1.87), 173	0.99 (0.83–1.55), 59	0.372	0.398
Glucose, mg/dL	110.5 (92–133), 140	102 (91–125), 57	0.263	0.328
Sodium, mmol/L	137 (134–140), 143	139 (136–140.75), 58	**0.010**	**0.016**
Potassium, mmol/L	4.0 (3.6–4.4), 119	4.1 (3.7–4.33), 48	0.951	0.951
INR	1.39 (1.22–1.65), 155	1.25 (1.11–1.41), 56	**<0.001**	**0.002**
RBC, ×10^12^/L	4.00 (3.45–4.39), 172	4.27 (3.72–4.62), 59	**0.010**	**0.016**
Hb, g/dL	11.35 (10.28–13.00), 172	12.70 (11.53–13.90), 58	**<0.001**	**<0.001**
Hct, %	35.0 (31.95–39.85), 172	38.45 (35.35–42.18), 58	**<0.001**	**<0.001**
WBC, ×10^9^/L	9.66 (7.88–13.20), 172	8.37 (6.75–10.40), 59	**0.007**	**0.016**
Neutrophils, %	80.5 (74.25–87.45), 171	75.1 (67.85–84.60), 59	**0.013**	**0.020**
Lymphocytes, %	11.1 (5.93–17.80), 170	14.8 (7.85–22.45), 59	**0.008**	**0.016**
Platelets, ×10^9^/L	270.5 (201.75–357.5), 172	245 (212.5–329), 59	0.309	0.356
ESR, mm/h	40 (22–71.5), 123	31 (17.5–63.75), 56	0.216	0.295

Data are presented as median (interquartile range [IQR]). The number of available observations (*n*) is reported for each variable because laboratory data were not complete for all episodes. Between-group comparisons were performed using the Mann–Whitney U test. To account for multiple laboratory comparisons, *p*-values were additionally adjusted using the Benjamini–Hochberg false discovery rate (FDR) procedure. ALT, alanine aminotransferase; AST, aspartate aminotransferase; INR, international normalized ratio; RBC, red blood cell count; Hb, hemoglobin; Hct, hematocrit; WBC, white blood cell count; ESR, erythrocyte sedimentation rate. The bold is to highlight the *p* is less than 0.05.

## Data Availability

The original contributions presented in this study are included in the article/Supplementary Materials. Further inquiries can be directed to the corresponding authors.

## Supplementary Materials

The following supporting information can be downloaded at https://www.mdpi.com/article/10.3390/cancers18203368/s1, Table S1: STROBE Statement—Checklist of Items That Should Be Included in Reports of Observational Studies.

## Abbreviations

The following abbreviations are used in this manuscript:

ALT	Alanine aminotransferase
AST	Aspartate aminotransferase
CI	Confidence interval
ESR	Erythrocyte sedimentation rate
FDR	False discovery rate
Hb	Hemoglobin
Hct	Hematocrit
INR	International normalized ratio
IQR	Interquartile range
OR	Odds ratio
RBC	Red blood cell count
TTF-1	Thyroid transcription factor-1
WBC	White blood cell count
