# Supplementary material for: Occult Malignancy Presenting as Pericardial Effusion: The Diagnostic Contribution of Pericardial Fluid Cytology
Source: Cancers (Basel). 2026 Oct 18;18(20):3368. doi: 10.3390/cancers18203368 (PMC13702510; doi:10.3390/cancers18203368)
Supplement: Supplementary file 1 [file cancers-18-03368-s001.zip › cancers-4634515-supplementary.pdf]

Occult Malignancy Presenting as Pericardial Effusion: The Di-agnostic Contribution of Pericardial Fluid Cytology

Table S1. STROBE Statement—Checklist of Items That Should Be Included in Reports of Observational Studies.

	Item No	Recommendation
**Title and abstract**	1	(*a*) Indicate the study's design with a commonly used term in the title or the abstract: Retrospective observational cohort study – Abstract, p. 2; Materials and Methods, p. 6.
		(*b*) Provide in the abstract an informative and balanced summary of what was done and what was found: Abstract – Page 2
**Introduction**		
Background/rationale	2	Explain the scientific background and rationale for the investigation being reported: Introduction, Sections 1.1–1.4, pp. 3–6
Objectives	3	State specific objectives, including any prespecified hypotheses: Materials and Methods – Page 8
**Methods**		
Study design	4	Present key elements of study design early in the paper: Materials and Methods – Page 6
Setting	5	Describe the setting, locations, and relevant dates, including periods of recruitment, exposure, follow-up, and data collection: Materials and Methods – Pages 6–8, no post-discharge follow-up was performed.
Participants	6	(*a*) Give the eligibility criteria, and the sources and methods of selection of participants. Describe methods of follow-up: Materials and Methods, pp. 6–7; Figure 1, p. 7. No post-discharge follow-up was performed.
		(*b*) For matched studies, give matching criteria and number of exposed and unexposed: Not applicable – this was not a matched study
Variables	7	Clearly define all outcomes, exposures, predictors, potential confounders, and effect modifiers. Give diagnostic criteria, if applicable: Materials and Methods – Pages 7–8
Data sources/ measurement	8*	For each variable of interest, give sources of data and details of methods of assessment (measurement). Describe comparability of assessment methods if there is more than one group: Materials and Methods – Pages 7–8
Bias	9	Describe any efforts to address potential sources of bias: Materials and Methods, pp. 6–8; Discussion, Section 4.4 (Strengths, Limitations, and Future Perspectives).
Study size	10	Explain how the study size was arrived at: Materials and Methods, pp. 6–7; Figure 1, p. 7
Quantitative variables	11	Explain how quantitative variables were handled in the analyses. If applicable, describe which groupings were chosen and why: Materials and Methods, p. 8; Results, Sections 3.1–3.7.
Statistical methods	12	(*a*) Describe all statistical methods, including those used to control for confounding: Materials and Methods, p. 8; analyses were unadjusted, with no multivariable confounder adjustment.
		(*b*) Describe any methods used to examine subgroups and interactions: Materials and Methods, p. 8; Results, Sections 3.3–3.7. No formal

		interaction analyses were performed.
		(*c*) Explain how missing data were addressed: Materials and Methods, p. 8; missing data were not imputed, and analyses used available observations.
		(*d*) If applicable, explain how loss to follow-up was addressed: Not applicable – no post-discharge follow-up was performed
		(*e*) Describe any sensitivity analyses: Materials and Methods, p. 8; Results, Section 3.1, p. 9. Sensitivity analysis restricted to the first eligible hospitalization per patient.
**Results**		
Participants	13*	(a) Report numbers of individuals at each stage of study—eg numbers potentially eligible, examined for eligibility, confirmed eligible, included in the study, completing follow-up, and analysed: Materials and Methods, pp. 6–7; Results, Section 3.1, pp. 8–9; Figure 1, p. 7.
		(b) Give reasons for non-participation at each stage: Materials and Methods, p. 6; Figure 1, p. 7.
		(c) Consider use of a flow diagram: Figure 1 – Page 7
Descriptive data	14*	(a) Give characteristics of study participants (eg demographic, clinical, social) and information on exposures and potential confounders: Results, Section 3.1 – Page 8-9
		(b) Indicate number of participants with missing data for each variable of interest: Materials and Methods, pp. 7–8; Results, Sections 3.1–3.7, pp. 8–17; Table 1.
		(c) Summarise follow-up time (eg, average and total amount): Not applicable – no post-discharge follow-up was performed
Outcome data	15*	Report numbers of outcome events or summary measures over time: Results, Sections 3.1–3.7, pp. 8–17; Figures 2–4; Table 1.
Main results	16	(*a*) Give unadjusted estimates and, if applicable, confounder-adjusted estimates and their precision (eg, 95% confidence interval). Make clear which confounders were adjusted for and why they were included: Results, Sections 3.3–3.7, pp. 10–17. Unadjusted odds ratios and 95% confidence intervals are reported; no multivariable adjustment was performed.
		(*b*) Report category boundaries when continuous variables were categorized: Not applicable to the main statistical comparisons; continuous variables were generally analyzed without categorization.
		(*c*) If relevant, consider translating estimates of relative risk into absolute risk for a meaningful time period: Not applicable
Other analyses	17	Report other analyses done—eg analyses of subgroups and interactions, and sensitivity analyses: Results, Section 3.1, p. 9 (sensitivity analysis); Sections 3.3–3.7, pp. 10–17 (subgroup and exploratory analyses).
**Discussion**		
Key results	18	Summarise key results with reference to study objectives: Discussion, Section 4.1, pp. 17–19; Conclusions, pp. 25–26.
Limitations	19	Discuss limitations of the study, taking into account sources of potential bias or imprecision. Discuss both direction and magnitude of any potential bias: Discussion, Section 4.4, pp. 22–25.
Interpretation	20	Give a cautious overall interpretation of results considering objectives,

		limitations, multiplicity of analyses, results from similar studies, and other relevant evidence: Discussion, Sections 4.1–4.4, pp. 17–25; Conclusions, pp. 25–26.
Generalisability	21	Discuss the generalisability (external validity) of the study results: Discussion, Section 4.4, pp. 22–25.
**Other information**		
Funding	22	Give the source of funding and the role of the funders for the present study and, if applicable, for the original study on which the present article is based: Funding statement, p. 26. No external funding was received.

*Give information separately for exposed and unexposed groups.

Note: An Explanation and Elaboration article discusses each checklist item and gives methodological background and published examples of transparent reporting. The STROBE checklist is best used in conjunction with this article (freely available on the Web sites of PLoS Medicine at http://www.plosmedicine.org/, Annals of Internal Medicine at http://www.annals.org/, and Epidemiology at http://www.epidem.com/). Information on the STROBE Initiative is available at http://www.strobe-statement.org.
